# (*E*)-5-Bromo-3-(2,6-dichloro­benzyl­idene)indolin-2-one

**DOI:** 10.1107/S160053680903270X

**Published:** 2009-08-22

**Authors:** Hongming Zhang, Haribabu Ankati, Shashidhar Kumar Akubathini, Ed Biehl

**Affiliations:** a Department of Chemistry, Southern Methodist University, Dallas, TX 75275, USA

## Abstract

The title compound, C_15_H_8_BrCl_2_NO, crystallizes with two independent mol­ecules in the asymmetric unit. The dihedral angles between the two aromatic rings are 62.74 (9) and 63.50 (6)° in the two independent molecules. In the crystal, the mol­ecules are connected by N—H⋯O hydrogen bonds, forming two centrosymmetric dimers.

## Related literature

For the pharmacological properties of 3-substituted indoline-2-ones, see: Andreani *et al.* (2006 ▶); Johnson *et al.* (2005 ▶); Sun *et al.* (2003 ▶). a series of 3-substituted indoline-2-one derivatives have been synthesized in our laboratory and their neuroprotective activities have been tested, see: Ankati *et al.*, (2009 ▶); Balderamos *et al.* (2008 ▶). For the structures of some of the derivatives, see: Zhang *et al.* (2008 ▶, 2009*a* ▶,*b* ▶).
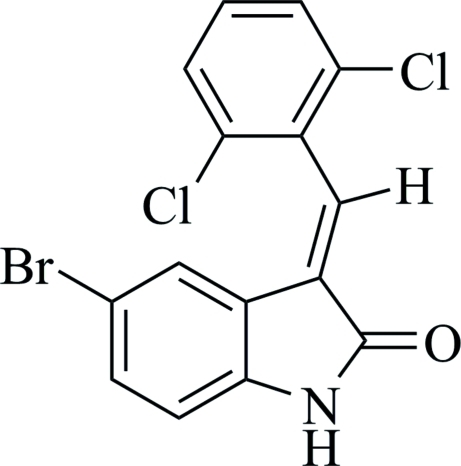


## Experimental

### 

#### Crystal data

C_15_H_8_BrCl_2_NO
*M* *_r_* = 369.03
Triclinic, 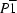

*a* = 8.1018 (4) Å
*b* = 13.5726 (7) Å
*c* = 14.4904 (7) Å
α = 63.575 (1)°
β = 82.956 (1)°
γ = 80.139 (1)°
*V* = 1403.89 (12) Å^3^
*Z* = 4
Mo *K*α radiation
μ = 3.30 mm^−1^
*T* = 296 K
0.32 × 0.21 × 0.15 mm

#### Data collection

Bruker SMART APEX diffractometer
Absorption correction: multi-scan (*SADABS*; Sheldrick, 1996 ▶) *T*_min_ = 0.416, *T*_max_ = 0.636
17925 measured reflections
6832 independent reflections
5263 reflections with *I* > 2σ(*I*)
*R*_int_ = 0.017

#### Refinement

*R*[*F* ^2^ > 2σ(*F* ^2^)] = 0.038
*wR*(*F* ^2^) = 0.100
*S* = 1.05
6832 reflections
361 parameters
H-atom parameters constrained
Δρ_max_ = 0.71 e Å^−3^
Δρ_min_ = −0.76 e Å^−3^

### 

Data collection: *SMART* (Bruker, 1997 ▶); cell refinement: *SAINT* (Bruker, 1997 ▶); data reduction: *SAINT*; program(s) used to solve structure: *SHELXS97* (Sheldrick, 2008 ▶); program(s) used to refine structure: *SHELXL97* (Sheldrick, 2008 ▶); molecular graphics: *SHELXTL* (Sheldrick, 2008 ▶); software used to prepare material for publication: *SHELXTL* and *publCIF* (Westrip, 2009 ▶).

## Supplementary Material

Crystal structure: contains datablocks I, global. DOI: 10.1107/S160053680903270X/bt5036sup1.cif

Structure factors: contains datablocks I. DOI: 10.1107/S160053680903270X/bt5036Isup2.hkl

Additional supplementary materials:  crystallographic information; 3D view; checkCIF report

## Figures and Tables

**Table 1:** Hydrogen-bond geometry (Å, °)

*D*—H⋯*A*	*D*—H	H⋯*A*	*D*⋯*A*	*D*—H⋯*A*
N21—H21⋯O22^i^	0.86	2.03	2.848 (3)	160
N1—H1⋯O2^ii^	0.86	2.09	2.924 (3)	163

Symmetry codes: (i) 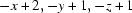; (ii) 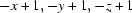.

**Fig. 1.**
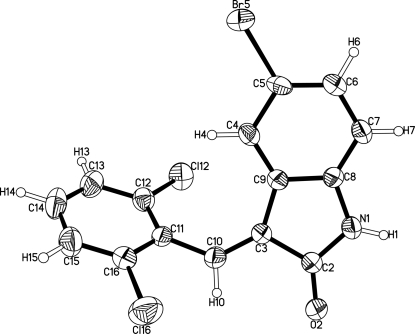
: A view of one of the independent molecules with displacement ellipsoids drawn at the 40% probability level. H atoms are presented as open circles with arbitrary radii. Atoms of another independent molecule were labeled as N21 H21 C22 O22 through C36 Cl36.

**Fig. 2.**
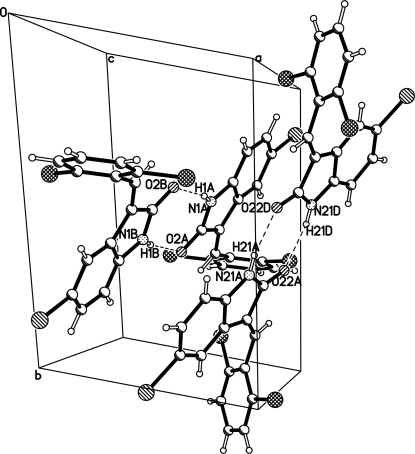
: A unit cell packing view of the title compound. Dash lines indicate hydrogen bonds. For clarity, H atoms are presented as open circles with arbitrary radii.

## supplementary crystallographic information

### Comment

3-Substituted indoline-2-ones have well recognized pharmacological properties, including antitumor properties (Andreani *et al.*, 2006), as well as the function as receptor tyrosine kinase (RTK) inhibitors (Sun *et al.*, 2003) and neuroprotective properties (Johnson *et al.*, 2005). For studying the biological properties a series of 3-substituted indoline-2-one derivatives have been synthesized in our lab and their neuroprotective activities have been tested (Ankati *et al.*, 2009, Balderamos *et al.* 2008). As a part of our research on the relationship between the biological activities and solid structures a couple of crystal structures of the derivatives have been carried out (Zhang *et al.*, 2008, 2009*a*, 2009*b*). The crystal structure confirmed the *E *configuration of the compound. The title compound consists of an oxindolyl and a dichlorophenyl unit (Fig 1). The dihedral angles between the two aromatic rings are 62.74 (9) and 63.50 (6)°, respectively, for the two independent molecules. The crystal structure revealed that the intermolecular H-bonds (Table 1), linking the two inverted molecules, form an octa cyclic membered ring and a dimer (Fig 2) is constructed.

### Experimental

2,6-Dichlorobenzaldehyde (1 mmol) was added to a mixture of 5-bromo-2-oxindole (1 mmol) and piperidene (0.1 mmol) in ethanol (4 ml) were placed in a microwave test tube. The tube was then capped and charged into a CEM microwave instrument. The mixture was irradiated with 250 psi pressure and at a temperature of 140°C for 10 minutes. Then the reaction mixture was left overnight at room temperature. The obtained solid was collected by filtration and washed with cold ethanol. The crude product was purified by recrystallization from ethanol and the yellow colored single-crystal sample was obtained.

### Refinement

All H atoms were placed in calculated positions and included in the final cycles of refinement using a riding model, with distances N–H = 0.86 Å and C–H = 0.93 Å, and displacement parameters *U*_iso_(H) = 1.2*U*_eq_(N,C).

### Crystal data

**Table:**

C_15_H_8_BrCl_2_NO	*Z* = 4
*M**_r_* = 369.03	*F*(000) = 728
Triclinic, *P*1	*D*_x_ = 1.746 Mg m^−^^3^
*a* = 8.1018 (4) Å	Mo *K*α radiation, λ = 0.71073 Å
*b* = 13.5726 (7) Å	Cell parameters from 8432 reflections
*c* = 14.4904 (7) Å	θ = 2.6–28.2°
α = 63.575 (1)°	µ = 3.30 mm^−^^1^
β = 82.956 (1)°	*T* = 296 K
γ = 80.139 (1)°	Rods, yellow
*V* = 1403.89 (12) Å^3^	0.32 × 0.21 × 0.15 mm

### Data collection

**Table:**

Bruker SMART APEX diffractometer	6832 independent reflections
Radiation source: fine-focus sealed tube	5263 reflections with *I* > 2σ(*I*)
graphite	*R*_int_ = 0.017
Detector resolution: 83.33 pixels mm^-1^	θ_max_ = 28.3°, θ_min_ = 1.7°
φ and ω scans	*h* = −10→10
Absorption correction: multi-scan (*SADABS*; Sheldrick, 1996)	*k* = −17→17
*T*_min_ = 0.416, *T*_max_ = 0.636	*l* = −19→19
17925 measured reflections	

### Refinement

**Table:**

Refinement on *F*^2^	Primary atom site location: structure-invariant direct methods
Least-squares matrix: full	Secondary atom site location: difference Fourier map
*R*[*F*^2^ > 2σ(*F*^2^)] = 0.038	Hydrogen site location: inferred from neighbouring sites
*wR*(*F*^2^) = 0.100	H-atom parameters constrained
*S* = 1.05	*w* = 1/[σ^2^(*F*_o_^2^) + (0.0447*P*)^2^ + 0.697*P*] where *P* = (*F*_o_^2^ + 2*F*_c_^2^)/3
6832 reflections	(Δ/σ)_max_ = 0.001
361 parameters	Δρ_max_ = 0.71 e Å^−^^3^
0 restraints	Δρ_min_ = −0.76 e Å^−^^3^

### Special details

**Table:**

Geometry. All e.s.d.'s (except the e.s.d. in the dihedral angle between two l.s. planes) are estimated using the full covariance matrix. The cell e.s.d.'s are taken into account individually in the estimation of e.s.d.'s in distances, angles and torsion angles; correlations between e.s.d.'s in cell parameters are only used when they are defined by crystal symmetry. An approximate (isotropic) treatment of cell e.s.d.'s is used for estimating e.s.d.'s involving l.s. planes.
Refinement. Refinement of *F*^2^ against ALL reflections. The weighted *R*-factor *wR* and goodness of fit *S* are based on *F*^2^, conventional *R*-factors *R* are based on *F*, with *F* set to zero for negative *F*^2^. The threshold expression of *F*^2^ > σ(*F*^2^) is used only for calculating *R*-factors(gt) *etc*. and is not relevant to the choice of reflections for refinement. *R*-factors based on *F*^2^ are statistically about twice as large as those based on *F*, and *R*- factors based on ALL data will be even larger.

### Fractional atomic coordinates and isotropic or equivalent isotropic displacement parameters (Å^2^)

**Table:**

	*x*	*y*	*z*	*U*_iso_*/*U*_eq_	
N1	0.6336 (3)	0.42436 (16)	0.61350 (15)	0.0430 (5)	
H1	0.6158	0.4089	0.5644	0.052*	
C2	0.5806 (3)	0.5237 (2)	0.61601 (18)	0.0405 (5)	
O2	0.4970 (3)	0.60288 (15)	0.55199 (14)	0.0556 (5)	
C3	0.6430 (3)	0.51560 (18)	0.71349 (17)	0.0360 (5)	
C4	0.8056 (3)	0.34403 (19)	0.85758 (18)	0.0385 (5)	
H4	0.8125	0.3773	0.9007	0.046*	
Br5	0.96940 (5)	0.15148 (2)	1.01371 (3)	0.07361 (13)	
C5	0.8708 (3)	0.2340 (2)	0.88452 (19)	0.0426 (5)	
C6	0.8650 (4)	0.1838 (2)	0.8206 (2)	0.0500 (6)	
H6	0.9128	0.1104	0.8402	0.060*	
C7	0.7889 (3)	0.2417 (2)	0.7280 (2)	0.0479 (6)	
H7	0.7835	0.2082	0.6849	0.057*	
C8	0.7211 (3)	0.35044 (19)	0.70100 (18)	0.0376 (5)	
C9	0.7300 (3)	0.40275 (18)	0.76408 (17)	0.0349 (5)	
C10	0.6107 (3)	0.60116 (19)	0.73755 (18)	0.0395 (5)	
H10	0.5468	0.6637	0.6914	0.047*	
C11	0.6620 (3)	0.61086 (17)	0.82716 (17)	0.0353 (5)	
C12	0.8280 (3)	0.60502 (19)	0.84706 (18)	0.0397 (5)	
Cl12	0.98958 (9)	0.57914 (6)	0.76720 (6)	0.05545 (17)	
C13	0.8707 (4)	0.6252 (2)	0.9265 (2)	0.0536 (7)	
H13	0.9828	0.6214	0.9374	0.064*	
C14	0.7468 (4)	0.6506 (3)	0.9884 (2)	0.0607 (8)	
H14	0.7750	0.6635	1.0420	0.073*	
C15	0.5811 (4)	0.6572 (2)	0.9723 (2)	0.0543 (7)	
H15	0.4968	0.6744	1.0145	0.065*	
C16	0.5418 (3)	0.6379 (2)	0.89234 (19)	0.0428 (5)	
Cl16	0.33175 (9)	0.64656 (8)	0.87280 (6)	0.0684 (2)	
N21	0.8680 (3)	0.62560 (16)	0.40279 (16)	0.0434 (5)	
H21	0.8925	0.5598	0.4065	0.052*	
C22	0.9185 (3)	0.65805 (19)	0.46956 (18)	0.0419 (5)	
O22	1.0070 (3)	0.60169 (14)	0.54175 (14)	0.0579 (5)	
C23	0.8454 (3)	0.77842 (18)	0.43524 (18)	0.0390 (5)	
C24	0.6607 (3)	0.9048 (2)	0.27961 (19)	0.0430 (5)	
H24	0.6485	0.9686	0.2900	0.052*	
Br25	0.44460 (5)	1.03332 (3)	0.11691 (2)	0.07282 (12)	
C25	0.5849 (3)	0.9036 (2)	0.19976 (19)	0.0474 (6)	
C26	0.6033 (4)	0.8103 (2)	0.1816 (2)	0.0521 (6)	
H26	0.5526	0.8127	0.1263	0.063*	
C27	0.6976 (3)	0.7130 (2)	0.2461 (2)	0.0479 (6)	
H27	0.7107	0.6497	0.2349	0.058*	
C28	0.7708 (3)	0.71278 (19)	0.32688 (18)	0.0395 (5)	
C29	0.7555 (3)	0.80790 (19)	0.34385 (18)	0.0384 (5)	
C30	0.8691 (3)	0.83197 (19)	0.48988 (19)	0.0422 (5)	
H30	0.9233	0.7892	0.5510	0.051*	
C31	0.8202 (3)	0.95013 (19)	0.46537 (18)	0.0400 (5)	
C32	0.8786 (3)	1.0363 (2)	0.37702 (19)	0.0431 (6)	
Cl32	1.01330 (10)	1.00841 (6)	0.28626 (5)	0.05811 (18)	
C33	0.8399 (4)	1.1464 (2)	0.3591 (2)	0.0504 (6)	
H33	0.8807	1.2019	0.2991	0.060*	
C34	0.7402 (4)	1.1731 (2)	0.4310 (3)	0.0565 (7)	
H34	0.7134	1.2470	0.4193	0.068*	
C35	0.6804 (4)	1.0910 (2)	0.5199 (2)	0.0547 (7)	
H35	0.6124	1.1090	0.5682	0.066*	
C36	0.7223 (3)	0.9814 (2)	0.5366 (2)	0.0448 (6)	
Cl36	0.65098 (11)	0.87932 (6)	0.65137 (6)	0.0638 (2)	

### Atomic displacement parameters (Å^2^)

**Table:**

	*U*^11^	*U*^22^	*U*^33^	*U*^12^	*U*^13^	*U*^23^
N1	0.0579 (13)	0.0407 (11)	0.0374 (10)	0.0041 (9)	−0.0129 (9)	−0.0243 (9)
C2	0.0470 (13)	0.0406 (12)	0.0382 (12)	0.0014 (10)	−0.0097 (10)	−0.0217 (10)
O2	0.0774 (13)	0.0466 (10)	0.0478 (10)	0.0152 (9)	−0.0307 (9)	−0.0262 (9)
C3	0.0416 (12)	0.0362 (11)	0.0320 (11)	−0.0009 (9)	−0.0080 (9)	−0.0162 (9)
C4	0.0453 (13)	0.0368 (12)	0.0361 (12)	−0.0017 (10)	−0.0087 (10)	−0.0179 (10)
Br5	0.1111 (3)	0.04428 (16)	0.0599 (2)	0.01126 (16)	−0.04425 (19)	−0.01523 (14)
C5	0.0495 (14)	0.0363 (12)	0.0382 (12)	0.0011 (10)	−0.0125 (10)	−0.0125 (10)
C6	0.0612 (16)	0.0339 (12)	0.0545 (15)	0.0061 (11)	−0.0119 (13)	−0.0208 (11)
C7	0.0598 (16)	0.0417 (13)	0.0503 (15)	0.0029 (11)	−0.0093 (12)	−0.0290 (12)
C8	0.0423 (12)	0.0374 (11)	0.0356 (11)	−0.0015 (9)	−0.0047 (9)	−0.0188 (10)
C9	0.0381 (12)	0.0322 (11)	0.0364 (11)	−0.0014 (9)	−0.0050 (9)	−0.0170 (9)
C10	0.0478 (13)	0.0337 (11)	0.0379 (12)	0.0021 (10)	−0.0131 (10)	−0.0161 (10)
C11	0.0459 (13)	0.0264 (10)	0.0342 (11)	−0.0025 (9)	−0.0085 (9)	−0.0130 (9)
C12	0.0464 (13)	0.0366 (12)	0.0374 (12)	−0.0046 (10)	−0.0031 (10)	−0.0171 (10)
Cl12	0.0489 (4)	0.0663 (4)	0.0600 (4)	−0.0104 (3)	0.0043 (3)	−0.0361 (4)
C13	0.0518 (15)	0.0670 (18)	0.0520 (15)	−0.0146 (13)	−0.0099 (12)	−0.0306 (14)
C14	0.074 (2)	0.076 (2)	0.0509 (16)	−0.0181 (16)	−0.0059 (14)	−0.0415 (16)
C15	0.0632 (18)	0.0613 (17)	0.0472 (15)	−0.0045 (14)	0.0003 (13)	−0.0334 (14)
C16	0.0465 (13)	0.0397 (12)	0.0430 (13)	−0.0008 (10)	−0.0082 (10)	−0.0188 (11)
Cl16	0.0448 (4)	0.0928 (6)	0.0682 (5)	0.0046 (4)	−0.0105 (3)	−0.0384 (4)
N21	0.0603 (13)	0.0286 (9)	0.0432 (11)	0.0016 (9)	−0.0053 (9)	−0.0194 (8)
C22	0.0593 (15)	0.0288 (11)	0.0356 (12)	0.0029 (10)	−0.0043 (11)	−0.0149 (9)
O22	0.0911 (15)	0.0341 (9)	0.0474 (10)	0.0165 (9)	−0.0246 (10)	−0.0201 (8)
C23	0.0516 (14)	0.0281 (10)	0.0367 (12)	0.0038 (10)	−0.0070 (10)	−0.0155 (9)
C24	0.0531 (14)	0.0336 (12)	0.0420 (13)	0.0012 (10)	−0.0065 (11)	−0.0176 (10)
Br25	0.0913 (3)	0.0635 (2)	0.05517 (19)	0.02261 (17)	−0.03042 (17)	−0.02304 (15)
C25	0.0557 (15)	0.0437 (13)	0.0381 (13)	0.0037 (11)	−0.0087 (11)	−0.0156 (11)
C26	0.0622 (17)	0.0571 (16)	0.0437 (14)	−0.0032 (13)	−0.0117 (12)	−0.0272 (13)
C27	0.0614 (16)	0.0445 (14)	0.0469 (14)	−0.0043 (12)	−0.0048 (12)	−0.0283 (12)
C28	0.0472 (13)	0.0340 (11)	0.0376 (12)	−0.0019 (10)	0.0003 (10)	−0.0177 (10)
C29	0.0479 (13)	0.0328 (11)	0.0353 (11)	−0.0007 (10)	−0.0034 (10)	−0.0170 (9)
C30	0.0563 (15)	0.0310 (11)	0.0379 (12)	0.0046 (10)	−0.0117 (11)	−0.0150 (10)
C31	0.0514 (14)	0.0311 (11)	0.0407 (12)	0.0032 (10)	−0.0158 (10)	−0.0182 (10)
C32	0.0559 (15)	0.0360 (12)	0.0414 (13)	0.0028 (11)	−0.0169 (11)	−0.0198 (10)
Cl32	0.0826 (5)	0.0496 (4)	0.0426 (3)	−0.0062 (3)	−0.0018 (3)	−0.0216 (3)
C33	0.0662 (17)	0.0316 (12)	0.0511 (15)	0.0004 (11)	−0.0242 (13)	−0.0129 (11)
C34	0.0621 (17)	0.0323 (12)	0.080 (2)	0.0102 (12)	−0.0234 (15)	−0.0300 (14)
C35	0.0555 (16)	0.0471 (15)	0.0724 (19)	0.0055 (12)	−0.0095 (14)	−0.0382 (15)
C36	0.0538 (15)	0.0372 (12)	0.0480 (14)	−0.0016 (11)	−0.0095 (11)	−0.0226 (11)
Cl36	0.0846 (5)	0.0547 (4)	0.0569 (4)	−0.0150 (4)	0.0079 (4)	−0.0293 (3)

### Geometric parameters (Å, °)

**Table:**

N1—C2	1.358 (3)	N21—C22	1.355 (3)
N1—C8	1.400 (3)	N21—C28	1.402 (3)
N1—H1	0.8600	N21—H21	0.8600
C2—O2	1.222 (3)	C22—O22	1.216 (3)
C2—C3	1.511 (3)	C22—C23	1.512 (3)
C3—C10	1.331 (3)	C23—C30	1.338 (3)
C3—C9	1.462 (3)	C23—C29	1.452 (3)
C4—C9	1.386 (3)	C24—C25	1.382 (4)
C4—C5	1.387 (3)	C24—C29	1.387 (3)
C4—H4	0.9300	C24—H24	0.9300
Br5—C5	1.895 (2)	Br25—C25	1.898 (3)
C5—C6	1.381 (4)	C25—C26	1.384 (4)
C6—C7	1.377 (4)	C26—C27	1.390 (4)
C6—H6	0.9300	C26—H26	0.9300
C7—C8	1.376 (3)	C27—C28	1.374 (3)
C7—H7	0.9300	C27—H27	0.9300
C8—C9	1.399 (3)	C28—C29	1.401 (3)
C10—C11	1.476 (3)	C30—C31	1.471 (3)
C10—H10	0.9300	C30—H30	0.9300
C11—C16	1.392 (3)	C31—C32	1.392 (4)
C11—C12	1.392 (3)	C31—C36	1.396 (3)
C12—C13	1.389 (3)	C32—C33	1.383 (3)
C12—Cl12	1.734 (2)	C32—Cl32	1.736 (3)
C13—C14	1.367 (4)	C33—C34	1.377 (4)
C13—H13	0.9300	C33—H33	0.9300
C14—C15	1.372 (4)	C34—C35	1.375 (4)
C14—H14	0.9300	C34—H34	0.9300
C15—C16	1.378 (4)	C35—C36	1.383 (4)
C15—H15	0.9300	C35—H35	0.9300
C16—Cl16	1.735 (3)	C36—Cl36	1.734 (3)
			
C2—N1—C8	111.37 (19)	C22—N21—C28	111.50 (19)
C2—N1—H1	124.3	C22—N21—H21	124.2
C8—N1—H1	124.3	C28—N21—H21	124.2
O2—C2—N1	126.1 (2)	O22—C22—N21	126.6 (2)
O2—C2—C3	127.3 (2)	O22—C22—C23	127.0 (2)
N1—C2—C3	106.56 (19)	N21—C22—C23	106.4 (2)
C10—C3—C9	134.0 (2)	C30—C23—C29	134.6 (2)
C10—C3—C2	120.7 (2)	C30—C23—C22	119.8 (2)
C9—C3—C2	105.30 (18)	C29—C23—C22	105.55 (19)
C9—C4—C5	117.6 (2)	C25—C24—C29	118.0 (2)
C9—C4—H4	121.2	C25—C24—H24	121.0
C5—C4—H4	121.2	C29—C24—H24	121.0
C6—C5—C4	122.1 (2)	C24—C25—C26	122.2 (2)
C6—C5—Br5	119.79 (18)	C24—C25—Br25	117.97 (19)
C4—C5—Br5	118.14 (18)	C26—C25—Br25	119.8 (2)
C7—C6—C5	120.4 (2)	C25—C26—C27	119.9 (2)
C7—C6—H6	119.8	C25—C26—H26	120.0
C5—C6—H6	119.8	C27—C26—H26	120.0
C8—C7—C6	118.2 (2)	C28—C27—C26	118.3 (2)
C8—C7—H7	120.9	C28—C27—H27	120.8
C6—C7—H7	120.9	C26—C27—H27	120.8
C7—C8—C9	121.8 (2)	C27—C28—C29	121.8 (2)
C7—C8—N1	128.7 (2)	C27—C28—N21	129.0 (2)
C9—C8—N1	109.52 (19)	C29—C28—N21	109.2 (2)
C4—C9—C8	119.9 (2)	C24—C29—C28	119.8 (2)
C4—C9—C3	132.8 (2)	C24—C29—C23	132.8 (2)
C8—C9—C3	107.23 (19)	C28—C29—C23	107.3 (2)
C3—C10—C11	129.2 (2)	C23—C30—C31	128.1 (2)
C3—C10—H10	115.4	C23—C30—H30	115.9
C11—C10—H10	115.4	C31—C30—H30	115.9
C16—C11—C12	115.5 (2)	C32—C31—C36	115.8 (2)
C16—C11—C10	120.2 (2)	C32—C31—C30	123.7 (2)
C12—C11—C10	124.0 (2)	C36—C31—C30	120.3 (2)
C13—C12—C11	122.2 (2)	C33—C32—C31	122.6 (2)
C13—C12—Cl12	117.7 (2)	C33—C32—Cl32	116.8 (2)
C11—C12—Cl12	120.01 (18)	C31—C32—Cl32	120.54 (18)
C14—C13—C12	119.5 (3)	C34—C33—C32	119.4 (3)
C14—C13—H13	120.2	C34—C33—H33	120.3
C12—C13—H13	120.2	C32—C33—H33	120.3
C13—C14—C15	120.6 (3)	C35—C34—C33	120.3 (2)
C13—C14—H14	119.7	C35—C34—H34	119.9
C15—C14—H14	119.7	C33—C34—H34	119.9
C14—C15—C16	118.8 (3)	C34—C35—C36	119.3 (3)
C14—C15—H15	120.6	C34—C35—H35	120.4
C16—C15—H15	120.6	C36—C35—H35	120.4
C15—C16—C11	123.3 (2)	C35—C36—C31	122.6 (3)
C15—C16—Cl16	118.3 (2)	C35—C36—Cl36	118.3 (2)
C11—C16—Cl16	118.34 (18)	C31—C36—Cl36	119.07 (19)

### Hydrogen-bond geometry (Å, °)

**Table:**

*D*—H···*A*	*D*—H	H···*A*	*D*···*A*	*D*—H···*A*
N21—H21···O22^i^	0.86	2.03	2.848 (3)	160
N1—H1···O2^ii^	0.86	2.09	2.924 (3)	163

Symmetry codes: (i) −*x*+2, −*y*+1, −*z*+1; (ii) −*x*+1, −*y*+1, −*z*+1.
